# Combination of polyglycerol sebacate coated with collagen for vascular engineering

**DOI:** 10.34172/jcvtr.2022.31

**Published:** 2022-09-06

**Authors:** Fateme Nazary Abrbekoh, Nasrin Valizadeh, Ayla Hassani, Hakime Ghale, Soltan Ali Mahboob, Reza Rahbarghazi, Ali Baradar Khoshfetrat, Mahdi Madipour

**Affiliations:** ^1^Department of Biochemistry, Higher Education Institute of Rab-Rashid, Tabriz, Iran; ^2^Department of Chemistry, Faculty of Science, Azarbaijan Shahid Madani University, Tabriz, Iran; ^3^Chemical Engineering Faculty, Sahand University of Technology, Tabriz, Iran; ^4^Department of Polymer Science and Engineering, University of Bonab, Bonab, Iran; ^5^Tuberculosis and Lung Disease Research Center, Tabriz University of Medical Sciences, Tabriz, Iran; ^6^Department of Applied Cell Sciences, Faculty of Advanced Medical Sciences, Tabriz University of Medical Sciences, Tabriz, Iran; ^7^Stem Cell Research Center, Tabriz University of Medical Sciences, Tabriz, Iran

**Keywords:** Polyglycerol Sebacate, Collagen, Endothelial Cells, Viability, Dynamic Growth

## Abstract

***Introduction:*** Here, we monitored the cytocompatibility of scaffolds consisting of poly (glycerol sebacate) (PGS) coated with collagen (Col) for endothelial cell activity after 72 hours.

***Methods:*** Human endothelial cells were allocated into Control, PGS, and PGS+Col groups. Scaffolds were characterized using FTIR and HNMR spectroscopy. Contact angel analysis and SEM were used to study wettability, surface morphology, and cell attachment. Cell survival was assessed using LDH leakage assay. Levels of Tie-1, Tie-2, VE-Cadherin, and VEGFR-2 were measured using western blotting and real-time PCR.

***Results:*** FTIR and HNMR analyses revealed the proper blending in PGS+Col group. SEM imaging exhibited a flat surface in the PGS group while thin Col fibers were detected in PGS+Col surface. The addition of Col to the PGS reduced the contract angle values from 97.3˚ to 81.1˚. Compared to PGS substrate alone, in PGS+Col group, cells appropriately attached to the surface. PGS and PGS+Col did not alter the leakage of LDH to the supernatant compared to control cells, showing the cytocopatiblity of PGS-based scaffolds. SOD and NO levels were increased significantly in PGS (*p*<0.05) and PGS+Col groups (*p*<0.001), respectively. We found that PGS+Col decreased Tie-1 content in endothelial cells whereas protein levels of Tie-2 and VE-Cadherin and expression of VEGFR-2 remained unchanged compared to PGS and control groups.

***Conclusion:*** Simultaneous application of Col and PGS can stimulate normal endothleial cell morphology without the alteration of tyrosine kinases receptors and cadherin.

## Introduction

 During the last decades, tissue engineering paves a way for the development of engineered scaffolds and hydrogel using *de novo* modalities for the treatments of several pathological conditions.^1-3^ Due to the lack of sufficient healthy autologous vessels, the development and fabrication of engineered vascular conduits are mandatory in patients requiring vascular bypass.^4^ On this basis, researchers have used a wide variety of substrates and biomaterials to mimic native extracellular matrix (ECM) structures and thrive the functionality of endothelial cells (ECs).^5^ The selection of appropriate biomaterials for the promotion of cell attachment, proliferation, and differentiation is the subject of debate.^6,7^ Besides, the biomaterials used for vascular engineering should possess suitable elasticity, withstand blood pressure, and lack thrombogenicity.^8-10^ To date, both synthetic and natural polymers have been used in the fabrication of engineered vascular tissues with varied advantages and disadvantages.^11^

 Col, especially type I, is the main component of the ECM and can interact with cell surface adhesion molecules, making this protein the most interesting ECM component in the fabrication of engineered transplants.^12^ Besides, the Col-based scaffolds are less invasive, biocompatible, and easily produced.^13^ Despite these advantages, less mechanical strength, high degradation rate, and instability at high temperatures have been shown in scaffolds consisting of Col.^14,15^ It seems that all mechanical properties can be improved by simultaneous application of synthetic and natural polymers.^16^

 PGS, a cytocompatible, hydrophobic, and synthetic polymer is used for the fabrication of several engineered scaffolds. Because of their suitable and unique physical properties, PGS-based scaffolds can be polymerized using different synthesis temperatures and reaction times.^17^ A plethora of experiments have used the combination of PGS with other substrates to reduce PGS hydrophobicity and enhance cell attachment.^18^ For instance, Rai *et al.* covered the PGS surface with fibronectin- and laminin-like polypeptides.^19^

 Here, we investigated the dynamic growth of human umbilical vein ECs (HUVECs) plated on PGS surface pre-coated with type I Col in *in vitro* condition. For this purpose, the survival rate was assessed using LDH leakage activity. Using Griess assay and monitoring the activity of GPx and SOD, nitrosative and oxidative stress was assessed. Protein levels and expression of Tie-1, -2, VE-Cadherin, and VEGFR-2 were measured using western blotting and real-time PCR analyses, respectively. Data from the current study can help us to understand molecular mechanisms which are associated with the regenerative potential of PGS-based scaffolds.

## Material and Methods

###  Materials

 Scaffold backbone was fabricated using pure Sebacic acid (Alfa-Aesar; Germany; purity 98%), Glycerol (purity 87%), Dimethyl Sulfoxide (DMSO, 99.0%), and EtOH were purchased from Merck (Germany). Pepsin enzyme (Sigma-Aldrich, Germany) and hydrochloric acid (Merck, Germany) were used for the extraction of type I Col. Penicillin-Streptomycin (Pen-Strep) and fetal bovine serum (FBS), High content glucose Dulbecco’s Modified Eagle Medium (DMEM/HG), phosphate-buffered saline (PBS), 0.25% Trypsin-EDTA were purchased from Gibco (USA). Human ECs (HUVECs; C554) were purchased from the Iranian National Cell Bank.

###  PGS synthesis and characterization

####  PGS synthesis

 Sebacic acid powder and glycerol were mixed at the molar ratio of 1:1 in a two-neck round bottom flask under an N_2 _atmosphere. The polycondensation procedure was continued by using the Dean-Stark apparatus under the inert condition at 110°C for 24 hours. Pre-polymer was precipitated at ethanol centrifugation. After that, the polymer was rinsed in deionized water and dialyzed. We poured purified polymer into a silicon mold and transferred it to a vacuum oven under 40 mTorr pressure at 120°C for 48 hours.

###  Fourier-transform infrared spectroscopy (FT-IR)

 To assess the chemical properties of synthetic PGS, we performed FT-IR spectroscopy using Bruker Tensor 27 Germany. PGS was combined with potassium bromide (KBr) powder to yield a KBr pellet. The spectrum was within the wavenumber range of 250- 4500 cm^-1^.

###  NMR analysis

^1^HNMR spectra were obtained after dissolving PGS samples in Deuterated chloroform (CDCl3) and analyzed by Bruker Spectrospin Avance 400 MHz.

###  Col extraction

 Bovine cutaneous tissues were prepared from a local slaughterhouse. The skin was sliced into small sizes and Col content (Type 1) was extracted using both enzymatic (pepsin solution) and chemical (Hydrochloric acid) methods as previously described by published protocols.^20^ By changing the pH of the solution and centrifugation, the Col content was precipitated. Here, we prepared 0.5-1 Wt% Col solution for different analyses. The pH of Col was set to 7 for scaffold fabrication.

###  Modification of PGS surface using Col

 To this end, the synthesized PGS were chopped into small pieces (20 µm in diameter) and placed at bottom of the culture plates. To coat the PGS surface with Col, Col samples were overlaid on PGS membranes and incubated at 37°C for 2 hours. Following the freeze-drying procedure, the PGS + Col scaffolds were used for different analyses.

###  Scanning electron microscope (SEM) analysis

 The feature and morphology of PGS were examined pre- and post-Col coating using SEM analysis. For this purpose, both PGS and PGS + Col membranes were chopped into 1 × 1 cm slices, coated with gold nanoparticles using a sputter coater scanning, and visualized Mira 3T Scan system. We also performed SEM imaging for the evaluation of cellular morphology plated on the PGS and PGS + Col surface.

###  Contact angle analysis

 Both PGS and PGS + Col membranes were prepared with the size of 1 × 1 cm. The surface wettability (contact angle value) was measured via DataPhysics OCA 15 Plus system. The machine consists of a motorized syringe that puts a drop of water on the designed scaffold. After placing a droplet on the sample surface, the contact angle was measured using the image analysis software (PGX, Thwing-Albert Instrument Co., USA).

###  Hydrolytic degradation analysis

 Again, membranes from both groups were prepared with the size of 1 × 1 cm. To calculate the biodegradation rate, samples were weighted (W₀) and placed in PBS at 37°Cwith gentle shaking for consecutive 25 days. The scaffolds were taken and freeze-dried every 5 days and weighted (W_1_) until the completion of the incubation period. Eventually, the percentage of polymer weight loss was calculated using the below formula


$$Weight\;loss\;\left(\%\right)=\frac{W_{0}-W_{1}}{W_{0}}\times 100$$

###  Cell culture 

 HUVECs were cultured in DMEM/HG culture medium supplemented with 10% FBS and 1% Pen-Strep. HUVECs were kept at 37°C with 5% CO_2_, and 95% relative humidity. At 70-80% confluence, cells were detached by using 0.25% Trypsin-EDTA solution and sub-cultured. HUVECs between passages 3-6 were subjected to different analyses. In this study, the cells were randomly allocated into three different groups as follows; Control; PGS, and PGS + Col. In the control group, cells were placed on the conventional plastic surface.

###  Lactate dehydrogenase (LDH) leakage assay 

 Membranes were cut into circles and placed on the bottom surface of 96-well plates (SPL). An initial number of 1 × 10^4^ HUVECs was suspended in 200 µl DMEM/HG medium containing 1-2% FBS and transferred into each well. After a 72-hour incubation time, the supernatant was collected and the level of released LDH was calculated using an LDH kit (PARS AZMON, Iran). The absorbencies were read at 360 nm using a Microplate Reader (ELx808; BioTek).

###  Measuring nitrosative stress using Griess reaction 

 The levels of nitrite oxide (NO) were measured 72 hours after the culture of HUVECs on the PGS and PGS + Col surfaces. In short, 200 µl DMDM/HG containing 1-2% FBS and 1 × 10^4^ HUVECs were transferred into each well of 96-well plates and kept for 72 hours. After that, 200 µl supernatant was mixed with 20 µl Griess A solution and maintained at room temperature for 10 minutes followed by the addition of 20 µl of Griess B solution. The samples were left for 2 minutes at room temperature and the OD of the samples was read at 540 nm. The contents of NO were calculated in all groups after comparison of OD values with the standard curve using sodium nitrite.

###  Measuring anti-oxidant capacity 

 In this regard, 6 × 10^5^ HUVECs were cultured on each well of 6-well plates. After 72 hours, the total protein content was extracted using 300 µl protein lysis buffer (NaCl, NP-40, SDS, and Tris–HCl). Then, the lysates were collected and centrifuged at 12000 rpm for 20 minutes. Using RANDOX reagents kit (Crumlin UK), the levels of superoxide dismutase (SOD) and glutathione peroxide (GPX) were measured in supernatants using Roche Hitachi 911 Chemistry Analyzer. The levels were expressed as IU/mg of total protein.

###  Western blotting

 The protein levels of Tie-1, Tie-2, and VE-Cadherin were assessed using western blotting. For this purpose, 10 µg of protein lysate was electrophoresed on 10% SDS-PAGE and transferred to polyvinylidene difluoride membranes. Then, membranes were blocked in 5% non-fat milk and incubated with anti-human Tie-1, Tie-2, and VE-cadherin antibodies at 4°C overnight. After serial TBST washes, the membranes were incubated with HRP-conjugated secondary antibodies for 1 hour at room temperature. Following TBST washes, the immunoreactive bands were detected using X-ray films and chemiluminescence ECL solution (Bio-Rad). The band relative intensity was calculated by ImageJ software (NIH), and the data were normalized to β-actin as a housekeeping protein.

###  Real-time PCR analysis

 HUVECs (1×10^6^) were cultured on PGS and PGS + Col scaffolds for 72 hours. Total cellular RNA was extracted using a Trizol kit (Cat. No: 0000115, Maxwell, South Korea) and reversed-transcribed into cDNA using a cDNA synthesis kit (YT4500, Yekta Tajhiz Azma). In this study, specific primer sets were designed using Oligo 7 software (Table 1) for VEGFR-2 and GAPDH genes. The expression of each gene was measured using SYBRGreen and Roche LightCycler 96 instrument. The PCR process was carried out in three steps for 50 cycles as follows; denaturation step at 95°C for 10 seconds, annealing step at 59°C for 30 seconds, and extension step at 72°C for 20 seconds. The expression of the target gene was calculated using the 2^-ΔΔCt^ method after normalization to the internal housekeeping gene GAPDH.

**Table 1:** Primer list

**Gene number**	**Accession number**	**Sequence (5**➔**3′)**	**Annealing temperature (°C)**
GAPDH	NM_001256799.3	Forward: TTGACCTCAACTACATGGTTTACA Reverse: GCTCCTGGAAGATGGTGATG	59
VEGFR-2	NM_002253.4	Forward: CCAGCAAAAGCAGGGAGTCTGT Reverse: TGTCTGTGTCATCGGAGTGATATCC	59

###  Statistical analysis

 Data are expressed as mean ± SD. We performed One-Way ANOVA with Tukey post-hoc to measure statistically significant differences between the groups using Graph Pad Prism (version 8). *P* < 0.05 was considered statistically significant. Three sets of experiments were conducted for each analysis otherwise mentioned.

## Results

###  Polymer and modified surface characterization

####  FTIR spectrum interpretation

**Figure 1:**
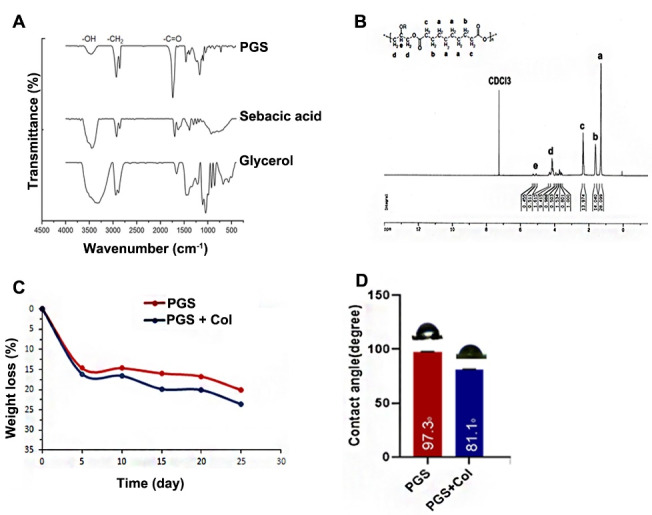
(A) PGS FT-IR Spectrum, (B) PGS NMR Analysis, (C) Comparing the PGS and PGS coated with collagen degradation rate, (D) The PGS surface successfully modified with collagen and the contact angle increased

 The FT-IR spectra confirmed that the PGS is synthesized correctly using the present protocol (Figure 1A). The carboxyl group (–COOH) of sebacic acid and hydroxyl group (–OH) of glycerol were combined during the polycondensation reaction and led to the formation of ester bonds in the PGS structure. FT-IR analysis of sebacic acid showed a peak at 1698 cm^-1^, indicating carboxyl (–COOH) group and alkyl (–CH2) bands at 2925 cm^-1^/2857 cm^-1^ and 1384 cm^-1^ (Figure 1A). In the sebacic acid group, the carboxylic acid O-H stretch band was detected around 3400 cm^-1^. FT-IR spectra of glycerol exhibited intense peaks at 1043 cm^-1^,2940 cm^-1^/2883 cm^-1^ and 3300 cm^-1^ associated with alkoxy group (C–O), alkyl groups (–CH2), and hydroxyl (–OH) of glycerol in the PGS structure (Figure 1A). Prominent peaks were shown at 1170 cm^-1^ and 2931 cm^-1^/2857 cm^-1^ correlate with carbonyl stretching bonds (C–O), and alkyl groups (–CH_2_), respectively. Also, the ester band (–COO) appeared at 1739 cm^-1^ in the PGS FT-IR spectrum.

###  HNMR spectrum analysis 

 HNMR analysis showed different peaks in the PGS structure during the polycondensation procedure (Figure 1B). The chloroform peak as the solvent was indicated at δ7.2 ppm. We noted eight protons of the sebacic acid appeared at δ1.2 ppm. Four protons were observed near and attached to the ester band in the sebacic acid section of the PGS structure at δ1.6 ppm and δ2.3 ppm, respectively. We assigned δ 4.1 ppm and δ 5.2 ppm peaks, indicating glycerol protons in the PGS structure.

###  Biodegradation assay 

 The biodegradation rate was calculated for both PGS and PGS + Col groups after a 25-day culture period (Figure 1C). According to our data, the mean weight loss for PGS was 16.08 ± 8.32% while this value reached 13.64 ± 6.97% in PGS + Col group. Results showed that the addition of Col to the PGS surface decreased the biodegradation rate.

###  Hydrophilicity of PGS surface is enhanced by Col 

 We performed contact angle analysis to determine surface wettability (Figure 1D). According to our data, the addition of Col to the PGS surface reduced the contact angle from 97.3˚ to 81.1˚, indicating an increase in surface wettability. Based on our data, the addition of Col increased surface hydrophilicity compared to the PGS group.

###  SEM analysis 

**Figure 2:**
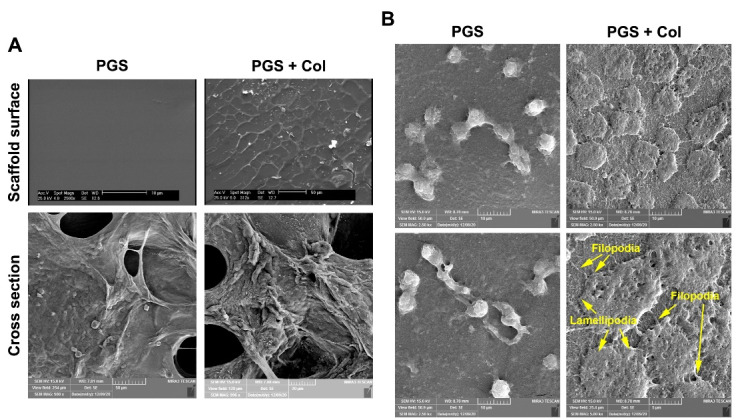
(A) The PGS surface modification by Col (B) The HUVECs confluence on the PGS and PGS surface modified by Col

 Monitoring the surface morphology by SEM imaging revealed that PGS exhibits a fairly flat and smooth surface (Figure 2A). In PGS + Col group, Col thin fibers generated grid and branch-like structures on the PGS surface. In cross-section analysis, both scaffolds possess a similar porous structure with an average pore size of 23.9 ± 12.6 µm (Figure 2A). We also cultured HUVECs on both surfaces and cell morphology was monitored. Based on data, we found that HUVECs did not attach appropriately to the PGS surface while these cells exhibited a flattened morphology in which cell filopodia and lamellipodia were detected (Figure 2B). It seems that the lack of appropriate cell attachment to the PGS surface may correlate with excessive hydrophobicity while the addition of Col supports cell attachment and adhesion. These data showed that the PGS + Col supports appropriately the adhesion and attachment of HUVECs compared to the PGS alone.

###  LDH leakage assay

**Figure 3:**
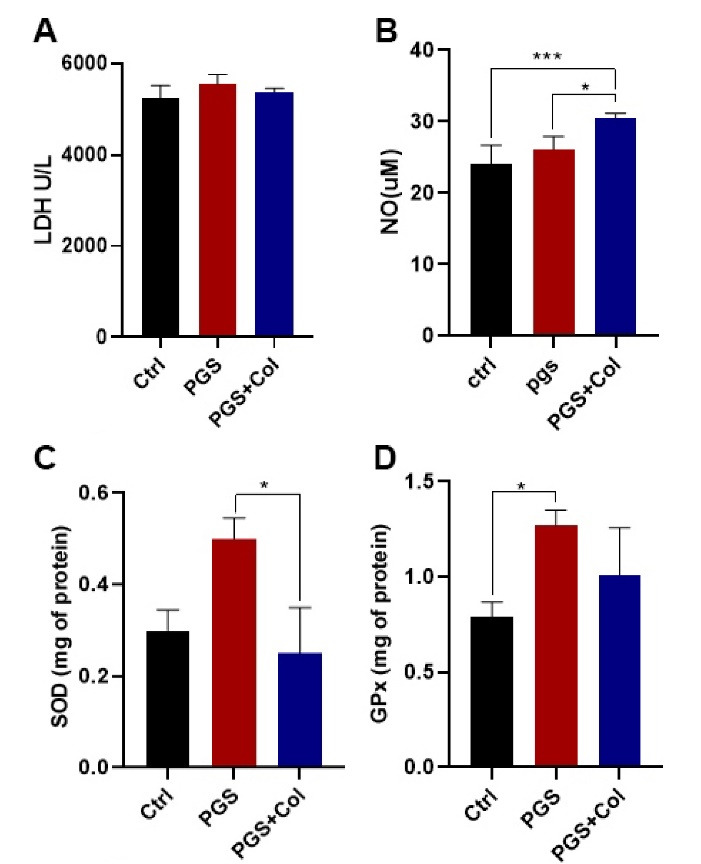
(A) LDH assay exhibited no significant difference between experimental groups regard to HUVECs viability after 72hrs in-vitro, (B) Evaluating NO levels using Griess assay after 72hrs (C; n = 3). According to our data, PGS + Col induces NO levels in HUVECS compared to the control group and PGS group, (C) Assessing oxidative stress utilizing RANDOX reagents kit 72hrs after HUVECs culture. Data pointed out that the PGS provokes oxidative stress in contrast to the control group and PGS + Col substrate. One-Way ANOVA assay with Tukey post hoc analysis. *p < 0.05; **P < 0.01; ***P < 0.001; ****P < 0.0001.

 LDH is a cytosolic enzyme and is released to the culture medium in after cell membrane injury ^21^. Here, we performed an LDH leakage assay to measure the HUVECs survival rate after 72 hours in PGS and PGS + Col groups (Figure 3A). Based on our data, the culture of HUVECs on the PGS and PGS + Col scaffolds did not yield statistically significant differences when compared to the control group (*P* > 0.05). These data showed that PGS-based scaffolds are suitable substrates for the culture and expansion of endothelial lineage.

###  NO levels were increased in HUVECs plated on PGS + Col surface

 The Griess method was used to measure NO levels in HUVECs after 72 hours. NO level was significantly increased in PGS + Col group compared to the control (*P* < 0.001) and PGS (*P* < 0.05) groups (Figure 3B). Data showed that the culture of HUVECs on the PGS surface did not significantly increase the NO levels compared to the control group (*P* > 0.05).

###  PGS, but not PGS + Col, induced oxidative stress 

 To measure oxidative stress, the activity of SOD and GPX were assessed (Figure 3C-D). Data showed that the culture of HUVECs on the PGS surface increased SOD activity compared to the PGS + Col group (*P* < 0.05). However, no statistically significant differences were obtained in the activity of SOD between the control and PGS groups (Figure 3C). Monitoring the levels of GPx revealed a significant increase in the PGS group compared to the control group (*P* < 0.05) (Figure 3D). Despite the increase of GPx in the PGS + Col group, we did not find statistically significant differences in GPx values between the control and PGS + Col groups. These data showed that the culture of HUVECs on the PGS, but not PGS + Col, induced oxidative stress compared to the conventional culture condition (Control group). We also noted that the addition of Col to the PGS surface reduced oxidative stress, showing the suitability of PGS + Col for the culture and expansion of HUVECs.

###  Tie-1 protein level was reduced in PGS + Col surface 

**Figure 4:**
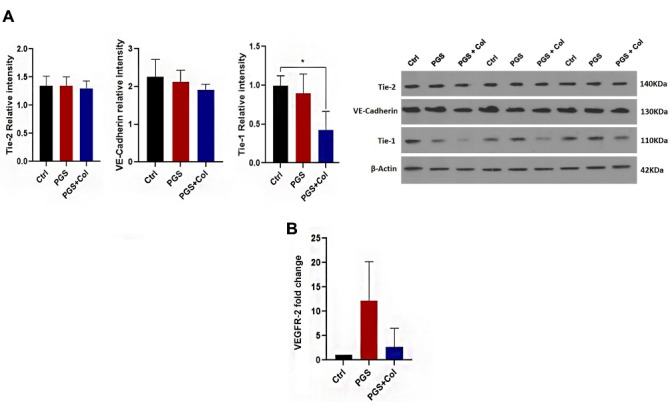
(A) Western blotting. Data showed no significant difference in Tie-2, VE-Cadherin, and β-Actin expression between groups. The Tie-1 expression decreased in HUVECs seeded on PGS + Col substrate (B) Real-time PCR analysis. Consider to our data, the VEGFR-2 expression enhanced in HUVECs seeded on PGS and PGS + Col insignificantly. One-Way ANOVA assay with Tukey post hoc analysis. *P < 0.05; **P < 0.01; ***P < 0.001; ****P < 0.0001.

 We measured the protein levels of Tie-1, Tie-2, and VE-Cadherin in HUVECs cultured on the PGS and PGS + Col surfaces (Figure 4A). Based on the data, we found that Tie-2 and VE-Cadherin levels were not changed significantly in PGS and PGS + Col groups when compared to the control group (*P* > 0.05). Of note, the culture of HUVECs on the PGS + Col surface reduced levels of Tie-1 compared to the control group (*P* < 0.05) while no statistically significant differences were obtained between the PGS and PGS + Col groups.

###  Real-time PCR analysis

 We conducted a real-time PCR assay to estimate the expression of VEGFR-2 in PGS and PGS + Col groups (Figure 4B). Despite the increase of VEGFR-2 expression in the PGS group, we found non-significant differences between all groups. Similar to changes in in PGS group, no statistically significant differences were found in the expression of VEGFR-2 between the control and PGS + Col groups. These data showed that the culture of HUVECs on PGS and PGS + Col surface did not alter the expression of VEGFR-2.

## Discussion

 In the fabrication of engineered vascular grafts, the combination of synthetic polymers and natural protein is somehow challenging.^22^ PGS, a hydrophobic, biodegradable polymer, is capable of altering the elasticity and hydrophobicity.^23^ It was suggested that the PGS possesses low molecular weight with less electrospinnability.^24^ In this regard, many studies have used various materials to manipulate the typical monomer ratios of PGS, and to change synthesis time to improve mechanical and physical features.^25,26^ Natural polymers such as Col can improve several features of synthesized scaffolds by the alteration of biocompatibility, hydrophilicity, and cell bioactivity.^27^

 In this study, we aimed to assess the dynamic growth of HUVECs after culture on the PGS pre-coated with the Col. Here, the decoration of PGS with type I Col was done at room temperature because high-temperature protocol, enzymatic digestion, UV irradiation, and photochemical interactions could damage Col structure and reduce natural bioactivity.^28^ We noted that the addition of Col to the PGS surface reduced the contact angle from 97.3˚ to 81.1, indicating increased hydrophilicity. The increase of wettability in the presence of Col is related to the existence of hydroxyl groups provided by Col on the PGS surface.^29^ Consistent with our data, Ravichandran, and co-workers reported the addition of Col to PGS in a core-shell fiber system can reduce the contact angle and reached 17˚.^30^ Based on previously published data, the culture of cells on a hydrophobic surface can limit dynamic growth, attachment of cells, and differentiation capacity.^31^ It was suggested that appropriate hydrophilicity can increase the biodistribution of oxygen and nutrients to deep layers of scaffolds.^31^ Along with this claim, SEM imaging showed promoted cell flattening in PGS + Col group compared to HUVECs plated on the PGS surface. We also found that the addition of Col to the PGS surface reduces the biodegradation rate from 87 to 84%. Numerous experiments have been conducted to reduce the biodegradation of PGS.^32^ Based on the LDH leakage assay, we found that PGS alone or in combination with Col did not exert cytotoxic effects on HUVECs after 72 hours. Our data also proved the cell compatibility of the PGS and PGS coated with Col. It has been shown that the co-culture of stem cells and cardiomyocytes on PGS + Col promoted proliferation and survival rates significantly compared to the Col and PGS groups.^13^ The levels of NO were increased in cells plated on PGS + Col substrate compared to Col and control groups. Regarding the lack of cytotoxicity indicated by the LDH leakage assay, it seems that PGS + Col contributes to the production of NO at physiological ranges which is vital for the angiogenic behavior of HUVECs.^33^ The culture of HUVECs on PGS alone induced oxidative stress indicated by SOD and GPx induction while no statistically significant differences were observed in the levels of GPx and SOD between PGS + Col and control groups. The protein levels of Tie-1, Tie-2, and VE-Cadherin were monitored in cultured cells after 72 hours using western blotting. It has been shown that angiopoietins (Ang-1 and Ang-2) and their receptors (Tie-1 and Tie-2) with tyrosine kinase activity have a critical role in the function of ECs.^34,35^ Ang-1 is commonly released by pericytes and binds to Tie-2 receptor, leading to vascular stability and barrier function. Upon the attachment of Ang-1 to the Tie-2 receptor, several downstream effectors such as the Ang-1/Tie-2/Akt signaling pathway are activated, resulting in the promotion of cytoskeletal proteins and VE-Cadherin.^36^ Unlike Tie-2, Tie-1 is an orphan receptor without the ability to attach angiopoietins. This protein can regulate the function of Tie-2 via close interaction.^36^ Besides Tie-1 and -2 receptors, other receptors such as VEGFR-2 participate in the formation of new vessels via the activation of PI3K, Akt, and MAPK.^34^ Data showed that the protein levels of Tie-2 and VE-Cadherin were not altered in HUVECs plated on PGS and PGS + Col surfaces. Of note, we found a significant decrease of Tie-1 in the PGS + Col group compared to the control. In an experiment conducted by Wang and colleagues, they found that the blend of PGS, type I Col, and silk fibroin acts as a natural endovascular mat for the dynamic activity of HUVECs.^37^ They also claimed that electrospun nanofibers consisting of PGS, type I Col and silk fibroin led to an appropriate cell-to-cell junction with less thrombogenic properties compared to hydrogel forms.^37^ Consistently, we noted that HUVECs plated on PGS surface coated with type I Col acquired flattened morphologies without changes in the content of Tie-2 and VE-Cadherin. One reason for the lack of significant changes in the levels of Tie-2, VEGFR-2, and VE-Cadherin would be that we did not use electrospun PGS and type I Col nanofibers for *in vitro* analyses. Wang and colleagues showed that the fabrication of PGS and Col blends nanofibers exhibited more angiogenic potential compared to hydrogel form.^37^ In addition, HUVECs were cultured on PGS and PGS + Col for a limited incubation time (72 hours). It seems that long-term culture of HUVECs can appropriately reflect the changes in the levels of factors and receptors involved in angiogenesis. One reason for the reduction of Tie-1 in PGS + Col group would be that the reduction of Tie-1 receptors occurred in response to enhanced angiogenesis behavior of cells plated on PGS + Col surface.^38^ Previous data indicated context-dependent activity of Tie-1 in ECs. To be specific, the content of Tie-1 is reduced in angiogenic tip cells while its levels is similar to Tie-2 in stalk and phalanx ECs.^38^ Therefore, it seems that a reduced Tie-1 level is compensatory response to regulate Tie-2 activity in ECs after plating on PGS + Col surface. However, numerous studies are mandatory to show the underlying mechanisms beyond the Tie-1 expression and angiogenesis behavior of cells in *in vitro* and *in vivo* conditions.

## Conclusion

 PGS + Col surface provides an appropriate condition for the attachment and activity of HUVECs. The addition of Col to the PGS surface improved the physicochemical property of PGS. Future investigations should focus on the discovery of different molecular pathways which are associated with the angiogenesis behavior of HUVECs.

## Acknowledgments

 Authors wish to thank the personnel of the Stem Cell Research Center for their help and guidance

## Funding

 This is a report of database from PhD thesis registered in Tabriz University of Medical Sciences with the number 62374.

## Ethical approval

 This study was approved by the local ethics committee of Tabriz University of Medical Sciences (IR.TBZMED.VCR.REC.1398.001)

## Competing interest

 The authors declare no conflicts of interest.
